# Dropout Deep Belief Network Based Chinese Ancient Ceramic Non-Destructive Identification

**DOI:** 10.3390/s21041318

**Published:** 2021-02-12

**Authors:** Jizhong Huang, Yepeng Guan

**Affiliations:** 1 Institute for the Conservation of Cultural Heritage, Shanghai University, Shanghai 200444, China; hjizhong@163.com; 2 School of Communication and Information Engineering, Shanghai University, Shanghai 200444, China

**Keywords:** dropout deep belief network, ancient ceramic, multi-spectral data, fractional order differential, non-destructive identification

## Abstract

A non-destructive identification method was developed here based on dropout deep belief network in multi-spectral data of ancient ceramic. A fractional differential algorithm was proposed to enhance the spectral details by making use of the difference between the first and second-order differential pre-process spectral data. An unsupervised multi-layer restricted Boltzmann machine (RBM) was employed to extract some high-level features during pre-training. Some weight and bias values trained by RBM were used to initialize a back propagation (BP) neural network. The RBM deep belief network was fine-tuned by the BP neural network to promote the initiative performance of network training, which helped to overcome local optimal limitation of the network due to the random initializing weight parameter. The dropout strategy has been put forward into the RBM network to solve the over-fitting of small sample spectral data. The experimental results show that the proposed method has excellent recognition performance of the ceramics by comparisons with some other ones.

## 1. Introduction

As one of the great treasures of world cultural heritage, ancient ceramics has many kinds, all of them having rich cultural connotations and high scientific and technological research value for the study of human civilization history. In the traditional identification method of the ceramics, the ceramics are identified mainly by means of an eye view, hand touch, and other sensory means from the aspects of shape, glaze color, and body of ceramics. Experienced experts provide recognition and conclusion through the personally empirical combination of vision and tactile sense. Since there is no standard for the classification of ceramics, it is difficult for people to unify the fault source in the dating and the authenticity discrimination of ceramics [1]. To overcome the limitations of personal experience, a lot of research on ceramics has been done. X-ray diffraction, scanning electron microscopy, and spectral analysis were usually carried out to discriminate ceramics [2,3,4,5,6,7,8]. Another research area is based on the manufacturing process of ceramics, including firing temperature, raw material treatment, and glaze formula [9,10,11,12,13]. Some researchers have studied the differences in element composition and other related information of ceramics from different origins and periods [14,15].

Certain machine learning methods such as pattern recognition and artificial intelligence [16,17,18,19,20] have been increasingly applied to the ceramic discrimination in recent years. A fuzzy clustering method [16] was selected to classify ceramic fragments according to the surface texture of ceramics. Cluster analysis requires high similarity within the same species, and it needs to initialize cluster centers in advance. Different cluster centers will lead to different clustering results, and so the classification stability is not ideal. Back propagation (BP) neural network [17] was used to perform data stream classification. Since BP neural network is prone to fall into local optimum due to different values of random initialization weight parameters, the overall classification performance is limited. The celadon classification model was established in [19] through a combination of random forest algorithm and Mahalanobis distance. Random forest is also prone to cause over-fitting due to multi-spectral data with anomaly to some extent. 

Some machine learning approaches [21,22,23,24,25,26,27] have been developed to improve classification performance further. Analysis of performance quality and tuning effects of the machine-learning techniques was presented in [21], in which Adaboost ensembles were more easily optimized than the other techniques. The performance of thirteen methods was investigated for modelling and predicting mortgage early delinquency probabilities [22]. Heterogeneous ensemble methods lead to other methods in the training, out-of-sample, and out-of-time datasets in terms of risk classification. A model called B2S has been proposed by gathering the advantages of the ensemble-based approaches to overcome the imbalance problem of the EOR dataset [23]. This provided the highest accuracy among the 10 models studied. 

Several feature ranking methods have been evaluated for improving RFBoost [24]. A version of RFBoost namely “RFBoost1” was proposed in [24]. A distributed kernel extreme learning machines algorithm was proposed in [25]. The distributed subnetwork was adopted to reduce the computational complexity. A dual-branch deep convolution neural network was proposed for Polarimetric SAR image classification in [26]. The extracted features were combined into a fully connected layer that shares the polarization and spatial property. An ensemble of online linear models was developed to make predictions in partial feature information in [27]. The online feature selection approach adopted a linear model as the base classifier.

Since most of the acquired ceramics are rare and precious material wealth, its structure is not allowed to be destroyed. A well-known method applied is called non-destructive testing (NDT) [28]. The NDT techniques do not permanently alter the physical characteristics of the tested product. Furthermore, the NDT techniques are used because they are cheap and fast, and also the evaluation of the quality of the product is instantaneous [29]. Hyperspectral imaging is a popular non-destructive testing technique widely used for discriminating the information of the ceramics [2,3]. However, the hyperspectral imaging technique depends on the material properties of the surfaces to be monitored and on the environmental conditions [30,31,32]. 

A non-destructive method for determining information of the ceramics is developed in this paper. A frame of non-destructive ceramic identification based on multiple sensors includes (1) data acquisition; (2) data processing; (3) classification recognition. In the signal acquisition step, a spectrometer used provides hundreds of bands in specific parts of the electromagnetic spectrum. In the second step, data processing aims to suppress the influence of noise and irrelevant information from the spectral data. The objective is to enhance the spectral information that affects the discriminating results as well as computational efficiency. Our experimental results show that the classification accuracy of the ceramics is only 84.52% without differential treatment (order 0), while the classification accuracy based on different fractional differential is significantly higher than that of order 0. When the fractional differential is 0.6, the classification performance is the best, reaching 93.7%. In classification recognition, unsupervised multi-layer restricted Boltzmann machine (RBM) is employed to extract the features of the high-level spectral data further. The mean correlation coefficient of spectral data between features before RBM is 0.8916, while the correlation coefficients after dimension reduction by the first and second RBM are 0.5365 and 0.3861, respectively. This indicates that the correlation between features and redundancy can be significantly reduced by RBM. The deep belief network has been used to classify ceramics from different dynasties. When the number of RBM is 2 with 100 hidden layer nodes and the dropout ratio is 0.5, the classification performance is optimal. The average classification accuracy of all the ceramics is 92.8%, and the accuracy of the Qionglai is the highest with 93.7%. 

**Figure 1:**
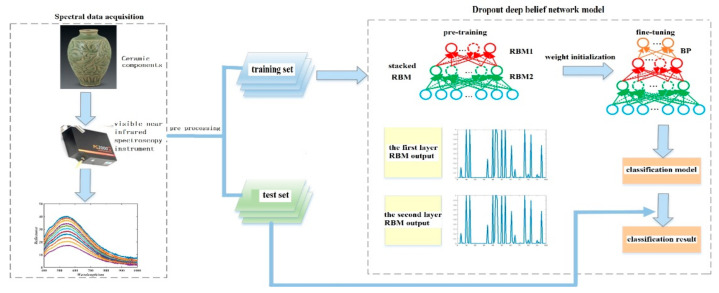
A flow chart of ceramic dating identification in multi-spectral data.

The main contributions are as follows. The first contribution of the developed method is that we propose a dropout deep-belief network to realize the non-destructive identification of ceramics. Dropout strategy has been put forward into a RBM deep belief network to solve the over-fitting of small sample spectral data. The second contribution is that we propose a fractional differential algorithm to enhance the spectral information by making use of the difference between the first-order and second-order differential spectral data. A third important contribution is to discriminate the ceramics from the spectral data without any hypothesis in advance. Comparison with some investigated methods indicates the superior performance of the proposal. A flow diagram is shown in Figure 1.

**Figure 2:**
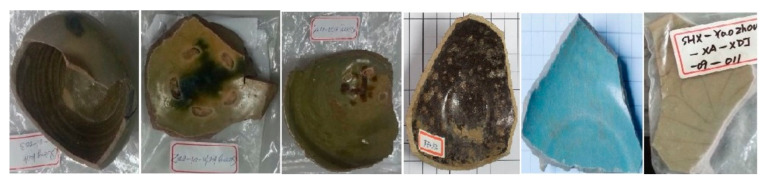
Some examples of the ceramic samples.

The spectral data of ceramics are acquired by a UV-VIS-NIR analyzer. Some ceramic samples are given in Figure 2

The spectral data are pre-processed based on fractional differential, and background information and noise interference are suppressed at first. Unsupervised learning RBM is selected to pre-train the deep network and to extract the features of the high-level spectral data. BP neural network is initialized with the weight parameters obtained from RBM pre-training, which are used to fine-tune the deep belief network. In addition, the deep belief network is fine-tuned to overcome the limitation of neural network falling into local optimal due to random initialization of weight parameters. In order to avoid over-fitting of small sample data in the training, a dropout random discard strategy is proposed to reduce the inter-dependence among the features. Both objective and quantitative comparisons are done with some methods. The experimental results show that the proposed method has excellent recognition performance of ceramics.

## 2. Experimental Data and Preprocessing

### 2.1. Spectral Data Acquisition

**Figure 3:**
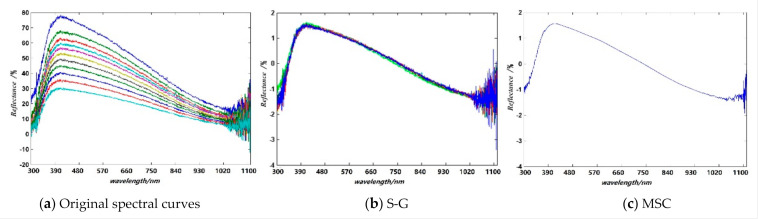
Some spectral curves of the ceramic from one kiln.

A PG2000-Pro UV-VIS-NIR spectrometer produced by Ideaoptics was adopted to collect the spectral data from different kilns. The incidence angle was measured at 10°–60° at intervals of 5°. The measurement angle was controlled by rotating a machine acquisition arm attached to a turntable whose rotation was controlled by a programmable motor. The reflection spectrum of the ceramic was obtained by measuring the ratio of reflected light at different incidence angles to the light source. The total number of valid sample data in the 300–1108 nm band was 3586, and each spectral data contained 1024 observed values. These samples included 638 pieces from Qionglai, 583 from Ru, 594 from Yaozhou, 572 from Guan, 583 from Yue, and 616 ones from Jun kilns in China. Some spectral curves of the sample from one kiln are shown in Figure 3.

It can be found from Figure 3 that the spectral curve has obvious burrs, especially at the range of 1000–1108 nm, which indicates there is a large spectral amplitude fluctuation anomaly. In order to get a reliable classification model of the ceramics, it is necessary to perform pre-processing as follows. In order to suppress the influence of noise and irrelevant information from the spectral data for the ceramics discrimination, Savitzky–Golay (S-G) filtering [33] was used to smooth the spectral data. The smoothed spectral curve is shown in Figure 3b. A multivariate scattering correction (MSC) [34] was selected to further smooth the spectral curve for avoiding the influence of uneven distribution on the sample surface. Some results are given in Figure 3c. It can be seen from Figure 3c that the difference between the spectral curves with different incident angles was significantly reduced after MSC. Even so, there is amplitude fluctuation anomaly seen, especially at the range of 1000–1108 nm still. In order to get a reliable classification of the ceramics, the visible-near-infrared band with a spectral wavelength range of 400–1000 nm is selected for analysis and discussion.

### 2.2. Pre-Processing Based on Fractional-Order Differential Spectrum

In the fields of traditional signal analyzing and processing, especially in singularities inspecting and extracting, integral differential-based algorithms have been wildly applied. However, the essence of modern signal analyzing and processing is to study the signals that feature non-linear, non-causal, non-minimum phase systems, non-Gaussian, non-even, nonintegral differential, and non-white additive noise [35]. Fractional differential is an effective mathematical method for dealing with fractal problems [36]. Many findings show that fractional-based algorithms are powerful approaches for dealing with the above-mentioned non-problems in signal processing [37,38,39]. In addition, fractional differential can be used for non-linearly enhancing complex fractal-like spectral data details [39,40]. Therefore, we could implement the Grümwald–Letnikov-based fractional differential.

**Figure 4:**
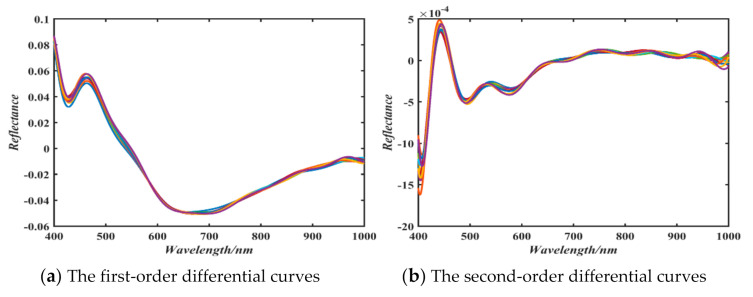
The first and second-order differentials pre-processing.

In order to further enhance the spectral information, the first and second-order derivatives of the spectral curves corrected by MSC are carried out. Some results are given in Figure 4.

The first-order derivative was selected to eliminate some linear noise and background information. The second-order differentials was used to eliminate the effects of baseline drift and background information. In addition, both the first and second-order differentials are used together to enhance spectral details. It can be seen from Figure 4 that the spectral curves of the first-order differential and the second-order differential differ greatly. In order to make use of the difference between first-order and second-order differentials, a fractional differential algorithm is proposed to pre-process spectral data.

Fractional differentiation is an extension of integer-order differentiation, which is used to study the mathematical properties and applications of differential mathematics of any order [41]. The fractional differential of Grunwald–Letnikov [42] is as follows:

(1) $$\frac{d^{\alpha }f(x)}{dx^{q}}=\underset{h\to 0}{\lim}\frac{1}{h^{\alpha }}\sum_{m=0}^{\frac{t-a}{h}}{\left(-1\right)}^{m}\frac{\Gamma (\alpha +1)}{m!\Gamma (\alpha -m+1)}f(x-mh)$$

where *α* is differential order, *h* is differential step length, *Г* is a gamma function, *q* is a real number, *f*(*x*) is a one-dimensional signal, *t* is a time variable of a one-dimensional signal, *m* ∈ *Z*, *Z* is a set of integers.

Since the sampling interval of spectral data is 0.6 nm, *h* in Equation (1) is set as 0.6 in the later experiment. The fractional differential of Equation (1) is expressed as following according to the limit theorem:

(2) $$w_{0}^{\left(\alpha \right)}=1;w_{m}^{\left(\alpha \right)}=\left(1-\frac{\alpha +1}{m}\right)w_{m-1}^{\left(\alpha \right)};\frac{d^{\alpha }f(x)}{dx^{q}}\approx \frac{1}{h^{\alpha }}\sum_{m=0}^{\frac{t-a}{h}}{\left(-1\right)}^{m}w_{m}^{\left(\alpha \right)}f(x-mh)$$

where $w_{m}^{\left(\alpha \right)}$ represents the coefficient after *m* iterations with *α* order.

**Figure 5:**
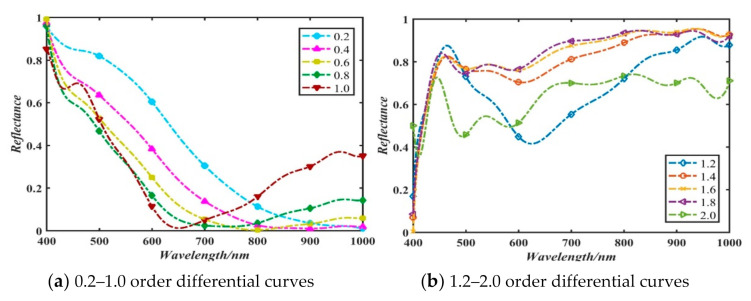
Different order differential processing of spectral data.

The differential results of different orders among 0.2–2.0 (interval is 0.2) can be obtained according to Equation (2). The data were mapped to 0–1 using the linear normalization method. Some results are shown in Figure 5.

It can be preliminarily found from Figure 5 that the spectral data have a gradual process with the increase of the differential order. How to discriminate the ceramics with different order differentials would be discussed and analyzed in subsequent experiments.

## 3. Classification of Ceramics Based on Dropout Deep Belief Network

### 3.1. Deep Belief Network

**Figure 6:**
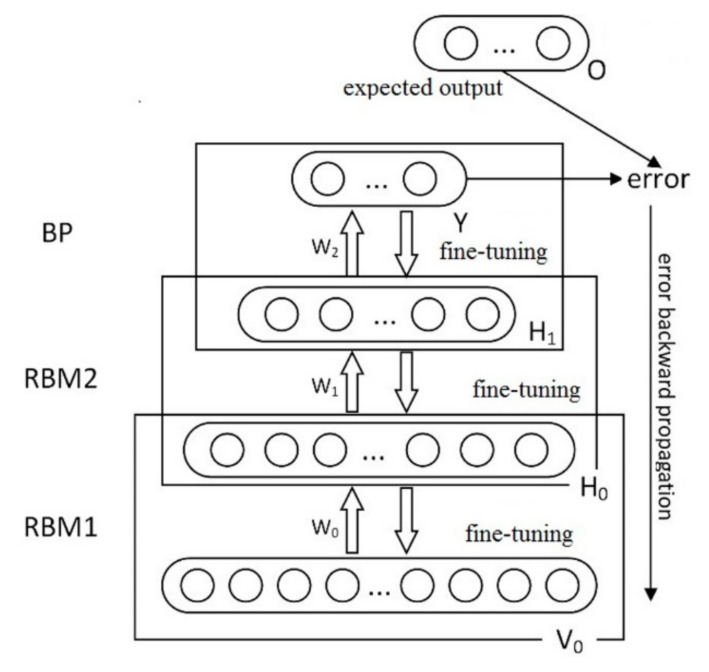
Deep belief network model diagram.

BP is a kind of widely used neural network model, which is often used in pattern classification. It is easy to fall into the local optimum due to its random initialization of weights and thresholds [43]. A deep belief network method for ceramics discrimination is proposed based on the RBM and BP neural networks. The network model is composed of a certain number of RBM and a layer of supervised BP back propagation network, shown as Figure 6.

The training of the deep belief network is divided into two stages: pre-training and fine-tuning to get good network parameters. The network adopts a step-by-step training strategy to carry out unsupervised training on each RBM layer during the pre-training. We first determine how the output of hidden layer *l* satisfies the following conditional probability according to the energy function of RBM and Bayesian equation [44]:

(3) $$p(l_{j}=1|v_{i})=\frac{1}{1+\exp(-bj-\sum_{i=1}^{m}w_{ij}v_{i})}=sigmoid(b_{j}+\sum_{i=1}^{m}w_{ij}v_{i})$$

where *w_ij_* represents the connection weight between visual layer unit *v_i_* and hidden one *l_j_*, *b_j_* is the bias of the hidden layer unit *l_j_*, *sigmoid* represents *S* function operator.

When the data of the output layer are determined, the conditional probability of the input visual layer element is as follows:

(4) $$p(v_{i}=1|l)=\frac{1}{1+\exp(-a_{i}-\sum_{j=1}^{n}w_{ij}l_{j})}=sigmoid(a_{i}+\sum_{j=1}^{n}w_{ij}l_{j})$$

where *a_i_* represents the bias of the visual layer unit *v_i_*.

Some redundant and irrelevant features will inevitably be introduced due to the high dimension of the original spectral data. In addition, some components may even play a negative role and bring about a dimension disaster. Both feature dimension and redundancy reduction strategies were adopted to overcome the limits as mentioned above. The deep belief network uses the stacked RBM structure to take the hidden layer output of the lower layer as the visible one input of the higher one. Some high level features of spectral data were extracted.

The following parameters were obtained by maximizing the logarithmic likelihood function of RBM on the input layer data *v* as:

(5) $$\frac{\partial \ln L(w,a,b)}{\partial wi_{j}}=p(l_{j}=1|v)v_{i}-\sum_{v}p(v)p\left(l_{j}=1|v\right)v_{i}$$

(6) $$\frac{\partial \ln L(w,a,b)}{\partial a_{i}}=v_{i}-\sum_{v}p(v)v_{i}$$

(7) $$\frac{\partial \ln L(w,a,b)}{\partial b_{j}}=p(l_{j}=1|v)-\sum_{v}p(v)p\left(l_{j}=1|v\right)$$

where *L*(*w*, *a*, *b*) represents the likelihood function with *w* connection weight, *a* and *b* biases.

The probability distribution *p*(*v*) requires a lot of calculation, and it cannot be calculated directly. The gradients in Equations (5)–(7) are approximately computed [45,46] as follows:

(8) $$a_{i}=a_{i}+\varepsilon \left[{v_{i}}^{\left(0\right)}-{v_{i}}^{\left(k\right)}\right]$$

(9) $$b_{j}=b_{j}+\varepsilon \left[p(l_{j}=1|v^{\left(0\right)})-P(l_{j}=1|v^{\left(k\right)})\right]$$

(10) $$\Delta w_{ij}=w_{ij}+\varepsilon \left[p(l_{j}=1|v^{\left(0\right)})vi^{\left(0\right)}-P(l_{j}=1|v^{\left(k\right)}){v_{i}}^{\left(k\right)}\right]$$

where *ε* is the learning rate, *v_i_*^(0)^ is the sample value, *v_i_*^(*k*)^ is the sample satisfying distribution *p*(*v*).

The supervised BP network is used for training during the fine-tuning. The back propagation of the BP network is used to return the error and to further fine-tune the weights and threshold parameters of the depth belief network so that the error between the actual output and the expected one can reach the minimum value.

### 3.2. Dropout Random Discard Strategy

**Figure 7:**
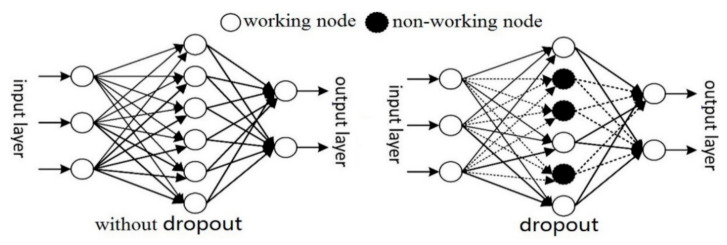
Dropout strategy in deep belief networks.

Deep neural network is mainly applied to a large number of data samples. For small sample data or samples with low characteristic dimensions, over-fitting may occur in the network. In order to prevent over-fitting, the following dropout strategy was developed in the deep belief network as follows. In the network training, the weights of some hidden layer nodes are randomly disabled with a certain probability *p* as shown in Figure 7.

The output of the *j*-th node in the hidden layer is as follows:

(11) $$l_{j}=X_{j}sigmoid(b_{j}+\sum_{i=1}^{m}w_{ij}v_{i})=\left\{\begin{array}{l} sigmoid(b_{j}+\sum_{i=1}^{m}w_{ij}v_{i}),\;\;\;\;\mathrm{if}\;\;X_{j}=1\\ 0\;\;\;\;\;\;\;\;\;\;\;\;\;\;\;\;\;\;\;,\;\;\;\;\mathrm{other}\;\mathrm{else}\;X_{j}=0 \end{array}\right.$$

where *P*(*X_j_* = 0) = *p*, *p* is the percentage of hidden nodes that are randomly discarded, *X_j_* represents whether the *j*-th node works or not.

Non-working nodes can temporarily be considered not part of the network structure. Its weight is retained, and it is only temporarily not updated. During the next iteration, these non-working hidden layer nodes may be re-used for training. In the case of small sample training data, too many iterations may lead to interdependencies among nodes. Some nodes in the hidden layer are randomly discarded during each iteration of dropout strategy, so the training network changes with each iteration to prevent interdependence between features.

## 4. Experimental Results and Discussions

### 4.1. Fractional Differential Order

**Figure 8:**
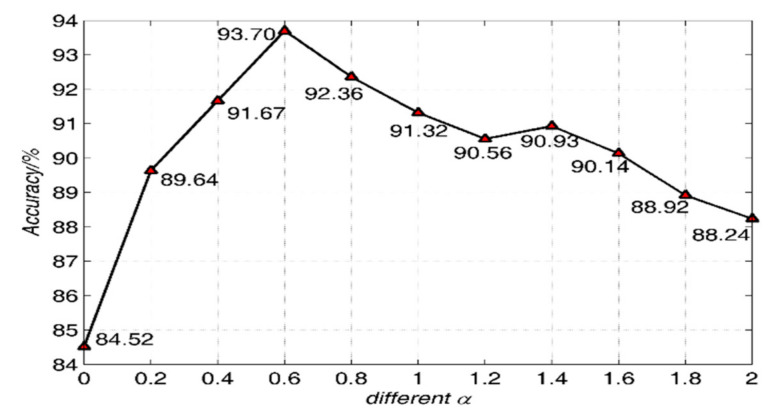
Classification performance of the ceramics with different α values for the Qionglai ceramics.

The α in Equation (2) is set as 0–2 with an interval of 0.2 for differential pretreatment, and the dropout-DBN deep belief network was used to classify the ceramics. The number of RBM layers was set as 2 layers, and the number of nodes in the hidden layers of RBM was fixed as 100. The result is shown in Figure 8.

It can be seen from Figure 8 that the classification performance of the ceramics improves with α increasing. When α is 0.6, the classification performance is the best. Then, classification performance decreases with α increasing. The performance is the lowest when α is 0, that is, no differential treatment is performed. The results show that fractional differential pretreatment is helpful to the discrimination of the ceramics. In the subsequent experiments, α in Equation (2) is set as 0.6 and keep the same.

### 4.2. Network Parameters

Since the range of the ceramic data used is 400–1000 nm and the sampling interval is 0.6 nm, the characteristic dimension of spectral data is 1000 ones. The number of units in the input layer of the first RBM in the dropout deep network was set as 1000. The number of units for hidden layer *H* and the number of RBM will be discussed later.

#### 4.2.1. RBM Number

In the dropout deep network, the number of stacked RBM affects the depth and classification performances. The higher RBM number is helpful to extract more high-level features for the classification performance. However, RBM makes the network depth deeper, the classification performance would be reduced [44,47,48]. On the one hand, the increase in the number of RBM means that more parameters are required to participate in the network model operation, which leads to the over-fitting of the network model. On the other hand, when the number of RBM increases, the errors caused by BP neural network for fine-tuning will be accumulated in the back propagation process.

In order to get the optimal number of RBM, the enumeration method was used to change the number of RBM from 1 to 6 in the experiment. The mean square error of the actual output *Y_k_* and the expected output *O_k_* is used for evaluation as:

(12) $$E=\frac{1}{2}\sum_{d=1}^{r}\sum_{k=1}^{q}(Y_{dk}-O_{dk}{)}^{2}$$

where *r* is the sample number, *q* is the node number of output layer.

**Figure 9:**
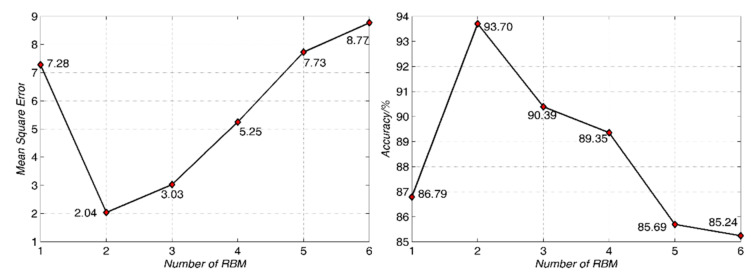
The output mean square errors and classification performances in different restricted Boltzmann machines (RBMs) for the Qionglai ceramics.

Some results for mean square error and overall classification accuracy for the ceramic are shown in Figure 9.

It can be seen from Figure 9 that the number of RBM has significant influence on the classification performance. When the number of RBM is 1, the mean square error is large, which indicates that the characteristics of RBM output layer cannot well fit the spectral data of input layer at this time. When the number of RBM increases to 2, the mean square error decreases significantly, and the classification performance reaches the optimal level. With the increase of RBM number, the mean square error increases gradually, and classification performance decreases gradually. The optimal number of RBM is 2. In the subsequent experiments, the number of RBM is set as 2 and keep the same.

#### 4.2.2. RBM Hidden Layer Node Number

**Figure 10:**
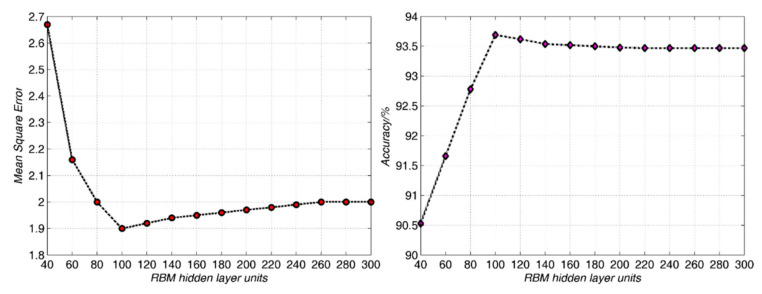
The output mean square errors and classifications in different RBM hidden layer nodes for the Qionglai ceramics.

The more nodes there are in the RBM hidden layer, the more accurate the distribution of training data can be represented by the RBM hidden layer [33,49]. The network may not be trained at all, or the network performance is poor if the number of hidden layer nodes is too small. However, if the number of nodes in the hidden layer is too great, the error would be increased. On the one hand, the training time of the whole network model would be prolonged. On the other hand, it is still easy to fall into the local minimum value instead of the optimal one. The number of hidden layer nodes must be far less than the number of training data, otherwise the network model has no generalization ability. In order to select the optimal number of hidden layer nodes, the enumeration method is selected to change the number of RBM hidden layer nodes from 50 to 300 with an interval of 20. The experimental results are shown in Figure 10.

One can find from Figure 10 that with the increase of the number of hidden layer nodes, the mean square error decreases gradually, and the classification performance increases gradually at first. When the number of hidden layer nodes is 100, the mean square error is significantly reduced, and the classification performance reaches the optimum. With the increase of the number of hidden layer nodes, the mean square error gradually increases, while the classification performance decreases gradually. It shows that when the number of hidden layer nodes is 100, the network performance is optimal. The number of nodes in RBM hidden layer is set as 100 and remains unchanged in the later experiment.

#### 4.2.3. Dropout Ratio

**Figure 11:**
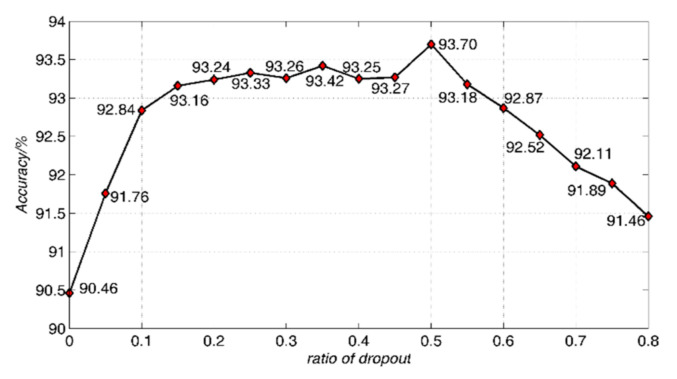
Some classification results in different dropout discard rates for the Qionglai ceramics.

The relationship between the proportion of randomly discarded hidden layer nodes and classification performance is shown in Figure 11.

It can be seen from Figure 11 that the classification performance is significantly improved after the dropout strategy. When the dropout ratio is 0.50, the classification performance is the highest with 93.7%. The classification performance decreases continuously with the increase of dropout discard ratio after 0.50. The dropout discard ratio is set as 0.50 and remains the same in the experiment.

### 4.3. Objective Quantitative Evaluation and Comparison

In order to further verify the fact that the stacked RBM can be used to eliminate some redundant features, Pearson correlation coefficient is selected to evaluate the performance as [50,51]:

(13) $$R=\frac{\left|\mathrm{cov}(X,Y)\right|}{\sqrt{\mathrm{var}(X_{i})\mathrm{var}(Y_{i})}}$$

where *cov* (*X*,*Y*) is the covariance of characteristic subset *X* and *Y*; *var* (*X_i_*) and *var* (*Y_i_*) represent the variances for *X* and *Y*, respectively.

The correlation between different features is lower if R in (13) is smaller, which indicates that the redundancy degree of the feature set is lower. The dimension of spectral data is 1000, and the size of the correlation coefficient matrix obtained is 1000 × 1000 before the characteristic dimension reduction. After the dimension reduction, the dimension is 100, and the size of the correlation coefficient matrix obtained is 100 × 100. Since the correlation coefficient matrix is large, the mean value and variance of correlation coefficients between statistical features are selected to perform comparison and analysis as shown in Table 1.

It can be found from Table 1 that the correlation between the features before dimension reduction (1000 dimensions) is as high as 0.8916, while the variance is only 0.0126. After dimension reduction by RBM in the first layer and the second layer, the correlation coefficient is reduced to 0.5365 and 0.3861, respectively. This indicates that the correlation between features is significantly reduced, and the data redundancy is significantly reduced compared with that before dimension reduction.

**Figure 12:**
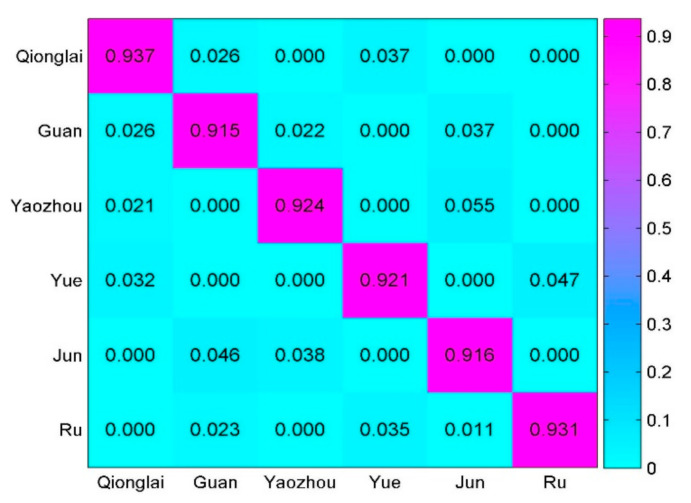
Classification performance of different kilns in confusion matrix.

Here, a 30-time 10-fold cross-validation was selected to perform a classification of the ceramics from six different kilns as mentioned above. In order to visually show the classification results, the confusion matrix was adopted, as shown in Figure 12.

The rows and columns of the confusion matrix represent the real category and the predicted category, respectively. Accuracy is calculated as the number of samples correctly predicted divided by the total number of samples.

In order to further evaluate whether the proposed method is effective to identify the ceramic from different kilns, the proposed method was compared with some other ones [16,17,19]. To get a fair test, a 30-time 10-fold cross-validation was done at the experiment. The algorithms used in [16,17,19] were used for experimental comparisons based on the obtained spectral data as mentioned above. The parameters used are all those recommended by the authors in [16,17,19], respectively. The experimental results are shown in Table 2.

One can find from Table 2 that the classification performance in our method is the best among the investigated methods [16,17,19]. The main reason is that the dropout deep belief network model proposed provides an appropriate initial value for BP neural network parameters through RBM unsupervised learning in the pre-training stage. The developed strategy avoids falling into the local optimum by random initialization weights of the BP network [17]. Fuzzy clustering [16] is prone to fall into the local optimum because of the fact that whether the cluster center value is appropriate. Different cluster centers would cause different clustering performances. Random forest [19] is also prone to cause over-fitting since the multi-spectral data obtained have obvious amplitude fluctuation anomaly, which is effectively solved by the developed pre-processing and the dropout deep belief network model.

## 5. Conclusions

A ceramic non-destructive identification method was proposed based on multi-spectral data. The spectral data are pre-processed in a fractional order differential way at first. The dropout deep belief network was adopted to realize classification of the ceramics. BP neural network was overcome in order to fall into the local optimum due to random initialization of the weight parameter. The dropout strategy was developed into a RBM deep belief network to solve the over-fitting of small sample spectral data. The experimental results show that the proposed method has excellent performance in the ceramics non-destructive identification.

## Figures and Tables

**Table 1:** Analysis of multispectral data before and after dimension reductions.

	Feature Dimension	Mean	Variance	Maximum	Minimum
before reduction	1000	0.8916	0.0126	0.9976	0.5154
first layer RBM	100	0.5365	0.0869	0.7763	0.0798
second layer RBM	100	0.3861	0.1256	0.5736	0.08687

**Table 2:** Experimental comparisons in different methods.

Method	Accuracy Rate (%)
Fuzzy clustering	85.8
BP network	86.3
machine learning	89.6
Ours	92.8

## Data Availability

Not applicable.
